# Supplementary material for: Student-, Study- and COVID-19-Related Predictors of Students’ Smoking, Binge Drinking and Cannabis Use before and during the Initial COVID-19 Lockdown in The Netherlands
Source: Int J Environ Res Public Health. 2022 Jan 12;19(2):812. doi: 10.3390/ijerph19020812 (PMC8776226; doi:10.3390/ijerph19020812)
Supplement: Supplementary file 1 [file ijerph-19-00812-s001.zip › ijerph-1531335-supplementary.pdf]

Supplemental files - Student-, study- and COVID-19-related predictors of students' smoking, binge drinking and cannabis use before and during the initial COVID-19 lockdown in the Netherlands

Authors names: Kirsten J.M. van Hooijdonk[1,*], Milagros Rubio[1], Sterre S.H. Simons[1], Tirza H.J. van Noorden[1], Maartje Luijten[1], Sabine A.E. Geurts[1], Jacqueline M. Vink[1]
[1]Behavioural Science Institute, Radboud University, Nijmegen, the Netherlands
*Corresponding author. E-mail address: kirsten.vanhooijdonk@ru.nl

Supplementary Materials S1

Detailed information on used scales and coding have been added below, per block of student-, study- and COVID-19-related characteristics.

Block 1: Demographics

Gender (male or female; category 'x' was disregarded due to the small amount N=76)), age (in years) and relationship status (steady relationship, single or complicated) of the participants was asked. Additionally, participants were asked if they were born in the Netherlands (yes/no). Educational level of mother and father was asked, where participants could report (1) less than secondary, (2) secondary, (3) higher education or (4) do not know. Category (4) was disregarded in the main analyses (N_{mother}=269, N_{father}=382). Last, participants were asked to state their living situation (before and during COVID-19). Answer categories included (1) with parents, (2) student hall, (3) accommodation with others, (4) alone, and (5) other.

Block 2: Study-related information before and/or during COVID-19

To assess study-related information, participants were asked in which type of study program they were enrolled during completing the survey, including (1) bachelor, (2) master, (3) PhD or (4) other, specify. If possible, participants who stated (4) other, specify… were recoded to one of the other categories. In the main analyses, categories (3) and (4) were disregarded (N=45). Additionally, students were asked whether this was their first year of education (yes/no). The importance of their study compared to other activities (before and during COVID-19) has been assessed by asking how important students found their studies compared to other activities, (1) more, (2) equally, (3) less. Regarding paying tuition, students could indicate how they covered the tuition of the current academic year. Students could select multiple answers including (1) not relevant, (2) parents paid, (3) paid myself, (4) bank or student loan, (5) scholarship, or (6) other, specify. Category (1) has not been included in the main analyses. If possible, participants who stated (6) other, specify… were recoded to one of the other categories. If it did not fit one of these categories the information was disregarded. Afterwards, dichotomous variables (yes/no) were created of categories 2-5. Next, participants were asked (before and during COVID-19) whether they agreed with the statement "I had sufficient financial resources to cover my monthly costs" on a Likert scale ranging from (1) strong agree to (5) strongly disagree. Last, students were asked (before and during COVID-19) how many hours per week they spent on (1) offline courses, (2) online courses, (3) personal study and (4) paid jobs. When the sum of these variables exceeded 150 hours all four sub-items were set to missing (N=2 sum sub-items >150 hours before and during, N=2 sum sub-items >150 hours only before, N=2 sum sub-items >150 hours only during).

Block 3: Study attitude factors during COVID-19

Study attitude (during COVID-19) was assessed by asking students to indicate on a 5-point Likert scale ((1) strongly disagree to (5) strongly agree) to which extent they agreed with

the following statements: “I work hard to succeed in my studies and spend a sufficient amount of time”, “Usually, I participate in all study activities” and “Being in touch with my fellow students helps me to perform well”.

Block 4: Study stress and well-being during COVID-19

Study-related concerns (during COVID-19) were assessed by asking participants, on a Likert scale ranging from (1) strongly agree to (5) strongly disagree, if they agreed with the following statements: “My university/college workload has significantly increased since the COVID-19 outbreak”, “I am concerned that I will not be able to successfully complete the academic year due to the COVID-19 outbreak”, and “The change in teaching methods resulting from the COVID-19 outbreak has caused me significant stress”. For the main analyses, these variables were reversely recoded, so that lower scores reflected lower stress levels and higher scores reflected higher stress levels. Student depressive symptoms (during COVID-19) was assessed by using the 8-item short version of the Center for Epidemiology Studies – Depression Scale (CES-D 8) (Karim et al., 2015). Cronbach’s alpha of the current study was 0.86. On a 4-point Likert scale ranging from (0) none or almost none of the time, (1) some of the time, (2) most of the time to (3) all or almost all of the time, participants were asked to indicate how much time during the past week … (a) “you felt depressed”, (b) “you felt that everything you did was an effort”, (c) “your sleep was restless”, (d) “you were happy”, (e) “you felt lonely”, (f) “you enjoyed life”, (g) “you felt sad”, and (h) “you could not get going”. A sum score of all 8 items was calculated (items (d) and (f) were reversely recoded) with higher sum scores reflecting greater depressive symptoms (range 0-24).

Block 5: Other factors related to the COVID-19 situation

To assess if the students had been infected with COVID-19, participants were asked if they had COVID-19 before or at the time of completing the survey. Answer categories included: (1) yes, confirmed by test, (2) yes, health care provider told me, (3) I think, but not confirmed or (4) no. Students degree of adherence to the COVID-19 regulations at the time of completing the survey was assessed on a Likert scale ranging from (0) totally not to (10) very strictly. Last, COVID-19-related worries were assessed on a scale ranging from 0 (not worried at all) to 10 (very worried). Depending on whether students had been officially diagnosed with a COVID-19 infection or not, students were asked to indicate their worries on the following statements: “How worried are you to get (re-)infected by COVID-19” and “How worried are you that you will get severely ill from a COVID-19 (re-)infection?”. Additionally, students were asked to indicate: “How worried are you that anyone from your personal network will get infected with COVID-19?”, “How worried are you that anyone from your personal network will get severely ill from a COVID-19 infection?” and “How worried are you that doctors and hospitals will not have sufficient medical supplies to handle the COVID-19 outbreak?”.

Supplementary Materials S2

Table S2. Univariate associations between the dichotomous main outcome variables (weekly smoking, weekly binge drinking, weekly cannabis use) and student-, study- and COVID-19-related factors as independent variables[#,§].

		**Weekly smoking**				**Weekly binge drinking**				**Weekly cannabis use**			
		Before		During		Before		During		Before		During	
1 - Demographics		OR	p	OR	p	OR	p	OR	p	OR	p	OR	p
Gender	Male* / Female	**0.59**	<.001	**0.58**	<.001	**0.53**	<.001	**0.46**	<.001	**0.29**	<.001	**0.38**	<.001
Age		**1.08**	<.001	**1.10**	<.001	**0.89**	<.001	1.03	.015	1.03	.028	1.02	.170
Country birth	Netherlands* / Other	**1.90**	<.001	**1.83**	<.001	**0.50**	<.001	0.98	.753	**3.09**	<.001	**2.71**	<.001
Education mother	(Less than) secondary* / higher education	0.97	.663	0.95	.451	1.02	.729	1.06	.304	**1.43**	<.001	**1.30**	.001
Education father	(Less than) secondary* / higher education	0.98	.742	1.05	.467	1.03	.583	0.91	.104	**1.40**	<.001	1.20	.019
Relationship status	Steady relationship*			**1**	<.001			**1**	<.001			**1**	<.001
	Single			0.90	.103			0.93	.255			**0.79**	.003
	Complicated			**2.05**	<.001			**1.95**	<.001			**2.25**	<.001
Living situation	With parent(s)*	**1**	<.001	**1**	<.001	**1**	<.001	**1**	<.001	**1**	<.001	**1**	<.001
	Student hall	0.98	.859	**1.67**	<.001	**1.70**	<.001	**2.74**	<.001	**2.55**	<.001	**3.99**	<.001
	With others	**1.57**	<.001	**2.59**	<.001	**2.17**	<.001	**2.83**	<.001	**3.26**	<.001	**4.78**	<.001
	Alone	**1.37**	.002	**2.79**	<.001	**1.35**	<.001	**2.00**	<.001	**2.63**	<.001	**4.58**	<.001
	Other	1.49	.014	**2.13**	<.001	1.03	.821	**2.12**	<.001	**2.51**	<.001	**3.78**	<.001
2 - Study-related information before and/or during COVID-19		OR	p	OR	p	OR	p	OR	p	OR	p	OR	p
Study program	Bachelor* / Master	**0.42**	<.001	**0.41**	<.001	0.92	.162	**0.75**	<.001	**0.72**	.001	**0.77**	.005
First year	Yes* / No	0.84	.054	0.86	.104	**1.21**	.004	1.13	.170	0.86	.212	0.92	.425
Importance study - other activities	More*	**1**	<.001	**1**	<.009	**1**	<.001	**1**	<.001	**1**	<.001	**1**	<.001
	Equally	**1.21**	.004	1.12	.088	**1.97**	<.001	**1.65**	<.001	1.24	.013	**1.29**	.001
	Less	**1.60**	<.001	**1.47**	.003	**2.71**	<.001	**2.37**	<.001	**1.80**	<.001	**1.80**	<.001
Tuition current year paid by…	Parents No* / Yes	1.05	.430	1.10	.138	**1.28**	<.001	1.11	.078	**1.47**	<.001	**1.30**	.001
	Myself No* / Yes	**0.74**	<.001	**0.74**	<.001	**0.78**	<.001	**0.82**	.004	**0.52**	<.001	**0.59**	<.001
	Loan No* / Yes	1.17	.019	1.11	.138	0.96	.375	1.08	.259	1.11	.242	**1.19**	.026
	Scholarship No* / Yes	0.74	.084	0.81	.202	**0.48**	<.001	**0.57**	.002	0.71	.141	0.82	.299
No sufficient financial resources[a]		**1.16**	<.001	**1.27**	<.001	**1.11**	.001	**1.25**	<.001	1.11	.045	**1.25**	<.001
How many hours spent on…	Offline course	**0.99**	.008	1.01	.115	**0.99**	<.001	1.00	.510	0.99	.046	0.98	.021
	Online course	1.01	.100	**0.99**	.005	**1.02**	.007	**0.98**	<.001	1.01	.477	1.00	.382
	Study time	**0.98**	<.001	**0.99**	<.001	**0.99**	<.001	**0.99**	<.001	1.00	.994	0.99	.075
	Paid job	**1.03**	<.001	1.01	.124	**1.02**	<.001	1.01	.070	1.00	.420	0.99	.013
3 - Study attitude factors during COVID-19				OR	p			OR	p			OR	p
I work hard to succeed in my studies and spend a sufficient amount of time				**0.83**	<.001			**0.82**	<.001			**0.82**	<.001
Usually, I participate in all study activities				**0.79**	<.001			**0.81**	<.001			**0.80**	<.001
Being in touch with my fellow students helps me to perform well				1.01	.670			1.01	.875			1.06	.128
4 - Study stress and well-being during COVID-19				OR	p			OR	p			OR	p
Workload increased				**1.16**	<.001			1.03	.246			1.08	.012
Concerned study success				**1.21**	<.001			**1.16**	<.001			**1.14**	<.001
Change in teaching causing stress				**1.22**	<.001			**1.09**	.001			**1.19**	<.001
Depressive symptoms				**1.07**	<.001			1.02	.011			**1.06**	<.001
5 - Other factors related to the COVID-19 situation				OR	p			OR	p			OR	p
COVID-19 infection	No*			1	.023			**1**	<.001			**1**	<.001
	Yes, confirmed by test			0.41	.387			0.77	.735			<.001	.998
	Yes, health care provider			1.45	.107			**1.73**	.009			**1.93**	.008
	I think so, but not confirmed			1.26	.010			**1.40**	<.001			**1.69**	<.001
Adhere to Covid-19 measures				**0.83**	<.001			**0.73**	<.001			**0.81**	<.001
COVID-19 related worries	(Re-)infection self			1.00	.886			**0.93**	<.001			1.00	.738
	Severely ill (re-)infection self			1.02	.117			**0.95**	<.001			0.98	.126
	(Re-)infection personal network			1.00	.958			**0.97**	.009			0.99	.498
	Severely ill (re-)infection personal network			1.02	.190			0.98	.060			1.00	.998

Sufficient medical supplies	1.00	.932	0.98	.089	1.02	.156

Note: Before refers to the month prior to the implementation of the first COVID-19 regulations. During refers to a week, prior to filling out the survey, during the first COVID-19 lockdown (April-July 2020).

* Reference category predictor.

[#] Significant predictors ($p<0.0083$) are presented in bold (Bonferroni correction 0.05/6).

[§] Gender and age were added as covariates to all univariate analyses.

[a] Higher scores indicate fewer financial resources.
